# Reconstructing Cross-Cultural Meanings of Addiction Among Women from Three Countries

**DOI:** 10.3390/ijerph22071064

**Published:** 2025-07-03

**Authors:** Caitlyn D. Placek, Lora Adair, Ishita Jain, Sugandh Gupta, Vandana Phadke, Maninder Singh

**Affiliations:** 1 Department of Anthropology, Ball State University, Muncie, IN 47306, USA; 2 Department of Life Sciences, Brunel University of London, London UB8 3PH, UK; lora.adair@brunel.ac.uk; 3 Department of Wellbeing, Regent College London, London WC1R 4BH, UK; ishita.jain@rcl.ac.uk; 4 Department of Anthropology, University of North Carolina, Chapel Hill, NC 27599, USA; sugandhgupta@ufl.edu; 5 Gait and Motion Analysis Lab, Center for Paediatric Orthopaedics and Disabilities, Gurugram 122002, India; vphadke@gmail.com; 6 Indian Spinal Injury Centre, Delhi 110037, India; drshah323@gmail.com

**Keywords:** culture, substance use disorders, hard-to-reach populations, free-listing, cultural domains

## Abstract

The gender gap in drug use is narrowing in regions where access to criminalized substances, such as opioids, is increasing. While research shows that substance use is gendered, less is known about the cultural norms and values shaping women’s drug use, as most studies focus on men. Cross-national comparisons of cultural models of addiction are needed to better understand how addiction is perceived and to inform culturally responsive treatment approaches for women. This study examined cultural models of addiction among reproductive-aged women receiving treatment for substance misuse in London, Toronto, and Delhi. Participants completed a semi-structured questionnaire with open-ended and free-list prompts. Findings revealed shared cultural models attributing drug use to psychological factors, such as self-medicating to manage negative emotions or enhance positive ones, as well as relational, developmental, and biological influences. In conclusion, the study highlights the importance of incorporating cultural models into research and treatment. By using an inductive approach to explore meanings surrounding drug use among people in recovery, researchers can better understand how interventions are received and interpreted through existing internal frameworks.

## 1. Introduction

Globally, the gender gap in drug use is closing in regions with increased access to criminalized drugs, such as opioids [1]. However, women’s drug use remains less understood due to cultural norms that frame drug use as a “man’s problem” [2,3,4,5]. In studies that include both men and women, men typically outnumber women in participation, and women tend to underreport their use [6,7]. Yet, drug use patterns and recovery seeking are gendered, with women navigating these experiences differently from men [8]. Understanding the cultural models that women use to guide their understanding and behaviors around substance use, addiction, and recovery is essential, especially as global substance use rates continue to rise and research gaps persist.

One pathway by which to understand women’s perceptions and experiences of drug use and addiction is via their internalized cultural models. Cultural models are a system of shared information and knowledge [9] that can be transmitted between individuals [10,11,12]. Culturally transmitted knowledge about illness often includes causal reasoning, a key component of medical knowledge, along with information about how to treat the illness [13,14]. In the southern United States of America (USA), for example, University students have been found to endorse cultural models of addiction that include both biological/medical (e.g., genetic predisposition, addictive properties of certain substances) and personal/social (e.g., boredom and curiosity, peer pressure, poor home life, low social class) causal attributions [15]. Causal models about health and illness are typically shaped by memories, a “pool” of cultural information, and social interactions [16,17]. Shared models of health and illness impact treatment-seeking behavior; to illustrate, in Guadalajara, Mexico, participants who shared the mainstream model of diabetes had better management of their condition [18].

Research highlights how cultural models of addiction can serve as both a deterrent and motivator of drug use and treatment seeking [19,20]. On one hand, people might avoid treatment if drug addiction is perceived as a moral failing [19,21]. Perceiving drug use as a moral failing also manifests into stigma, increased social distancing (e.g., avoiding treatment and recovery services due to previous experiences of social exclusion), and discrimination, and undermines efforts to increase access to care [15,19,22]. On the other hand, perceiving drug use as a chronic, relapsing brain disease is associated with more public support for medical treatment and access to medications for substance use disorders [23,24]. Explicit cultural models of addiction, however, have not been explored in a cross-cultural context among women.

Cross-country comparisons of cultural models of drug use and addiction are necessary to advance our understanding of how addiction is perceived and to guide collaborative efforts in developing therapeutic approaches for women from diverse cultural backgrounds who need treatment. Studies suggest that while addiction and recovery are often viewed as individual processes, such as abstinence for recovery [25], cultural perspectives reveal that these concepts are cross-culturally diverse and encompass social and structural dimensions [26]. A cross-cultural approach creates space for women to express their understandings of addiction and recovery in ways that are meaningful within their own cultural and social contexts, rather than solely through the dominant biomedical perspective. While biomedical models, such as the Brain Disease Model of Addiction (BDMA), focus on neurobiological explanations and individual pathology, they often fail to capture the diverse ways people make sense of their experiences. These models can overlook the social, structural, and cultural factors that profoundly influence how addiction and recovery are lived and understood. By engaging with cultural models across different settings, researchers and practitioners can uncover the values, meanings, and community dynamics that shape recovery in ways that may not align with biomedical paradigms. This approach highlights locally relevant definitions of recovery, particularly those that resonate with women within specific cultural or societal frameworks. Therefore, the present study aimed to examine cultural patterns of addiction among women of reproductive age receiving substance abuse treatment in London, Toronto, and Delhi.

## 2. Study Populations

This study examined cultural models of addiction in three regions—Toronto, Canada, London, England, and Delhi, India—where illicit drug use, specifically opioids, is rising. While public statements affirm officials’ commitment to reducing substance use in all three countries [27,28,29,30], they differ significantly in political approaches and policy responses, which may influence systemic and therapeutic solutions.

The Canadian government’s strategy is designed to reduce substance use-related harms and to reduce rising rates of overdose across the country. Specifically, this strategy has included increasing funding for community and grassroots programs designed to tackle the precursors of substance use (e.g., gang involvement, poor mental health, criminal behavior), increasing awareness of substance-use-related harms and reducing drug-use-related stigma through educational campaigns, and reducing drug-use-related harms through the provisioning of drug testing services (e.g., to see if drugs contain other harmful substances), access to clean needles and other drug use equipment, and supervised drug use sites (e.g., medical staff present who can intervene in case of accidental overdose; [31]). In some provinces, legislative change has resulted in the decriminalization of small amounts of certain drugs (e.g., heroin, cocaine, methamphetamine; [32]).

While substances like heroin and methamphetamine have not been decriminalized at a legislative level in the UK, police are increasingly directing drug-related offenders into education and treatment (in lieu of prosecution) as part of the government’s 10-year commitment to reduce drug use [33]. Stringent drug policies remain in place, with certain drugs (labeled “class A”) like heroin, cocaine, and ecstasy punishable by as much as seven years in prison (for possession) or even life imprisonment (for distribution). The UK government’s strategy to reduce substance use primarily exists at the intersection between criminal activity and drug use; for example, strategies include routine drug testing upon arrest and testing prison wastewater for the presence of drugs. According to a recent House of Commons Committee report [33], the primary institutional barrier to the reduction in substance use in the UK is funding, with annual spending on drug treatment services being cut by 40% between 2014 and 2022—while the Department of Health and Social Care has recently increased funding allocation to drug treatment services provided through local authorities, this has not restored funding to previous levels.

India enforces strict drug laws under the Narcotic Drugs and Psychotropic Substances (NDPS) Act, 1985, which prohibits the manufacture, sale, purchase, and possession of narcotics like heroin, cocaine, and synthetic drugs (except bhang). The Narcotics Control Bureau (NCB) actively monitors trafficking, particularly given India’s proximity to major opium-producing regions like Afghanistan and Myanmar. National and local government strategies such as the Nasha Mukt Bharat Abhiyan or the Drug-Free India Campaign are in place in India to reduce substance use rates and related harms, but they are severely limited in their coverage and reach due to a lack of trained professionals to implement substance use prevention programs, irregular implementation of government schemes, and negative public perception towards substance users [34]. For example, opioid agonist therapies are estimated to reach only about 4% of intravenous opiate users, and an individual user can only access about 34 needles/syringes through exchange programs per year [35,36].

Despite differences in drug legality, women in all three regions face barriers in disclosing substance use and seeking treatment, particularly during their reproductive years. A key deterrent is social stigma [37,38,39,40]. Women who use illicit drugs often experience guilt and shame, further discouraging them from seeking help [37]. Intersectional identities, such as motherhood, minoritized status, and sexually transmitted infections, exacerbate stigma for women with substance use disorders. This heightened stigma can deter them from seeking care, adhering to medication, and may even increase their risk of sexual health conditions [39,41]. For example, women in London and the northeast of England identify stigma and negative stereotyping as significant barriers to care [38,42,43]. In Delhi, women who use drugs often avoid sexual healthcare due to stigma from providers [39]. In Toronto, women who use opioids sometimes face violence linked to stigma surrounding their substance use disorder [44].

In summary, while the three countries differ in terms of laws surrounding substance use, they are similar in terms of rising opioid misuse and the social consequences of using illicit drugs. However, cultural models of addiction among women in these three regions have not been studied.

## 3. Materials and Methods

Data for this qualitative study were collected at different times in Toronto, Delhi, and London, regions chosen for their rising rates of substance misuse, particularly opioids, including heroin and fentanyl [45,46]. The study received approval from Ball State University’s Institutional Review Board on 28 July 2023 (#2068016), the Ethics Committee at the Indian Spinal Injuries Centre (1565696-4, 7 April 2021), and Brunel University of London’s College of Health (45852-NER-Nov/2023-47888-1), Medicine, and Life Sciences Research Ethics Committee. Data collection in Delhi took place from 2020 to 2021, while data collection in Toronto occurred from October to December 2023, and in London from January to March 2024.

Participants were initially recruited using convenience sampling through the directors of treatment centers, who were asked to share study information with eligible individuals. Due to COVID-19 restrictions in Delhi, interviews were conducted primarily online or by phone. For participants in Toronto and London, data collection took place in person or over the phone with women aged 18 and older. The inclusion criteria specified women aged 18 or older who were receiving treatment for a substance use disorder, with a focus on mothers in Toronto and London to explore their recovery experiences. We excluded participants who were actively engaged in substance use but not seeking treatment. We did not, however, exclude participants based on length of time in treatment or by drug of choice. Semi-structured interviews were pilot tested with two to three participants per group to ensure questions were interpreted similarly across sites. Participants responded to open-ended questions covering various topics related to substance addiction and recovery, including their perceptions of the causes of addiction, their recovery experiences, including the types of services they received and perceptions of those services, perceptions of harm reduction, and community perceptions of women and mothers who used substances, including barriers they faced in accessing addiction treatment. Interviews lasted between 20 and 60 min. All interviews were audio-recorded and transcribed. Participants received compensation following local norms. This study reports explicitly on participants’ perceptions regarding the causes of addiction.

## 4. Analysis

Data generated from the interviews were a mix of open-ended responses and free lists. Combining free-listing [47,48] with open-ended responses helps identify key themes and offers detailed insights into their meanings and significance. This study applied this hybrid method by asking participants to list perceptions of addiction (both their perceptions of addiction as well as those espoused within their community), with opportunities to expand on their reasoning, which enriched the contextual understanding of relevant cultural domains.

Each transcript initially underwent two iterations of coding; in the first iteration, the theme, “causes of addiction,” was identified for questions associated with addiction causes. For the second iteration of coding, the first author recoded sections labeled “causes of addiction” to inductively identify more specific themes related to perceived causes of addiction, such as “psychological,” “interpersonal factors,” and “genetics.” Next, themes related to the causes of addiction were identified in each excerpt, in the order they were mentioned, which allowed us to conduct a salience analysis [47,48]. Themes were recorded for consistency across participants. For example, when asked to free-list the causes of addiction, one participant in Delhi responded:

“Stress; Capability of not coping with what’s going on around you.”

The themes “stress” and “coping” emerged from this excerpt and were placed in a file with participant IDs in the first column, and themes across subsequent columns in the order participants mentioned them (e.g., Column B “stress” and Column C “coping”). Then, a salience score was calculated for each cause of addiction based on its order and frequency of mention. Salience analysis is a method used to identify the main items in a domain by computing a score for each item that reflects the frequency and order of mention [47,48,49]. The cut-off for salience in each population is determined by a Smith S score of >0.10 [50]. Below are results with a Smith S score ≥0.10 across each time point. Data were analyzed using a salience analysis with the AnthroTools package in R. All names have been changed to protect the identities of participants.

## 5. Results

A total of *n* = 49 women in Delhi, *n* = 19 women in Toronto, and *n* = 10 women in London completed the semi-structured in-depth interview guide. Differences in sample size were due to the nature of the study design and time constraints. In Delhi, for example, we had a year allocated for data collection, and qualitative data were paired with quantitative surveys due to COVID-19 restrictions. In Toronto and London, qualitative interviews preceded quantitative surveys, and data collection was limited to two months in each location. Therefore, our goal for Toronto and London was to achieve thematic saturation through qualitative surveys before conducting quantitative research, which was accomplished with these sample sizes. Once thematic saturation was achieved, results could be meaningfully compared across the three sites.

Respondents were all in recovery at treatment centers that ranged from non-residential, drop-in services to residential programs. The average age of women in Delhi was 23.9 (range = 18 to 41), in Toronto, 38.05 (range = 26 to 52), and in London, 42.11 (range = 35 to 52). In Delhi, 26 women had completed high school, and 16 had received a college education. The remaining participants (*n* = 7) did not report their educational attainment. In London, nine women had completed upper secondary education, two held a professional diploma, one had earned a master’s degree, and one reported no formal education. In Canada, one woman had completed middle school, eight had completed high school, and ten had received a college-level education. Participants also varied in their status as mothers. In London, the average number of children was 2.1 (range = 0–5), with one participant reporting no children. In Toronto, the average was 1.8 children (range = 0–5), and in Delhi, women had an average of 0.57 children (range = 0–3). None of the participants were pregnant at the time of the study.

The three regions overlapped yet varied in the types of drugs that women reported using. In all areas, women reported using heroin. In Delhi, other drugs included cannabis and hash. In Toronto, the drugs of choice were alcohol, cannabis, methamphetamine, fentanyl, benzodiazepines, psilocybin, and crack. In London, additional substances included alcohol, cannabis, cocaine, crack, ecstasy/MDMA, and spice.

### 5.1. What Causes Addiction?

#### 5.1.1. Delhi

**Figure 1:**
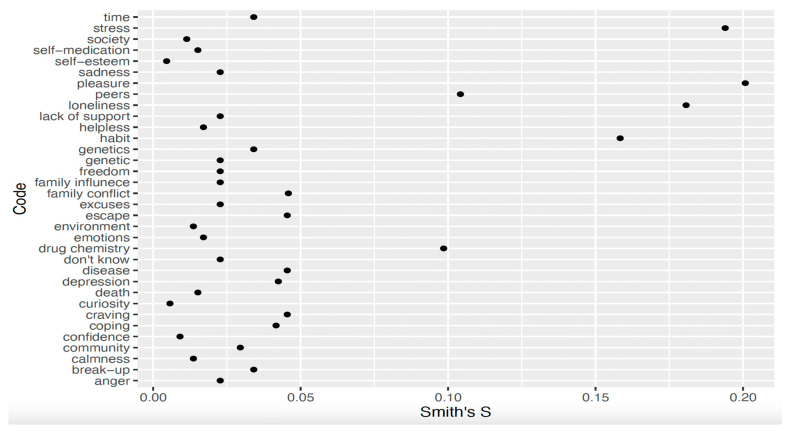
Smith’s S scores for perceived causes of addiction in Delhi.

In Delhi, the salient perceived causes of addiction for women in treatment were *pleasure* (Smith’s S = 0.20), *stress/tension* (Smith’s S = 0.19), *loneliness* (Smith’s S = 0.18), *habit* (Smith’s S = 0.16), and *peers* (Smith’s S = 0.10; Figure 1).

*Pleasure* (Smith’s S = 0.20) was described as a key antecedent of addiction, with participants positioning substance use in the context of recreation and having fun. Substance use was understood as a strategy to elicit positive feelings, which, over time, was characterized by the management or avoidance of negative feelings and sensations. For example, one participant, Selma, described addiction as grounded in pleasure and withdrawal avoidance: “They feel nice and relaxed after doing it; and then when they don’t use it; then they feel some pain in their body, so they keep using.” Other participants combined pleasure with other feelings; Aayana said, “30–40% start because of fun, but then 60% are those in emotional need and support, and then they select the wrong path and eventually get addicted. Max, a person can use for 2 years unless they are rich because after that, you get short on money and you get addicted as well, so it’s a whole spiral situation.” Overall, participants believed that addiction was caused, at least in part, by a desire to feel pleasure and avoid pain

*Stress/tension* (Smith’s S = 0.19) was also often mentioned as a cause of addiction and was typically a stand-alone cause. For example, Saanvi said that addiction was caused “by overdoing it. Tensions.” Another participant, Aditi, said, “Because of tension and stress, you take drugs and you feel de-stressed and then it becomes a habit.” In this example, Aditi acknowledges that habit is a secondary cause of addiction but is first dependent on the experience of stress. Similarly, another participant, Divya, stated, “Tensions. I had family tensions, and I stopped going to school as well.”

Descriptions of *loneliness* (Smith’s S = 0.18) as a cause of addiction included narratives of isolation, the death of someone close, separations, break-ups, and divorces; for example, Noor said, “Well, I got addicted due to loneliness, then there was my friend circle.” Another participant, Amya, mentioned loneliness first and then stress, or tension, as a secondary cause: “I think, lack of attention and constant tension.” Similarly, Jiya mentioned, “Everybody has their issues, some people are sad, or alone, and that’s why they do it.” Within these two cultural domains (stress and loneliness), we can see that substance use as a tool to manage psychological discomfort (caused by a variety of contextual and interpersonal processes) is viewed as precipitating addiction.

*Habit* (Smith’s S = 0.16) was described as the frequent and escalating use of a substance. This is summarized by Amara, who said, “When you take it once, and it becomes a habit, then it becomes an addiction, it becomes a need, you require it every day to feel what you felt in the first place.” Similarly, when describing the cause of addiction, Devika said, “Maybe because of regular usage.” As such, frequent use was viewed as a risk for developing unhealthy and addictive patterns of (mis)use.

*Peers* (Smith’s S = 0.10) was a theme that captured participants’ experiences with friends and acquaintances who introduced them to drugs. When Aashvi was asked about the cause of addiction, she said, “I think it was because of my friend group.” Peers, however, were not the highest in salience, as is evident from Smith’s S scores. As a result, it was often mentioned after other causes had been highlighted. A good example is from Aachal, who said, “I think people who get addicted are the cause of their addiction. They make reasons for themselves to get addicted, like a breakup, divorce, or family stress. People find reasons to take drugs. They also get influenced by people around them and what they see around them. Some people want to explore new options. This is what happened with me also.” Overall, participants saw peers (and interpersonal influences) as a key contributor to addiction but initially placed responsibility at the feet of the individual, attributing their initial patterns of frequent or habitual use to a desire to feel pleasure, avoid pain, or otherwise manage difficult situations or emotions.

#### 5.1.2. Toronto

**Figure 2:**
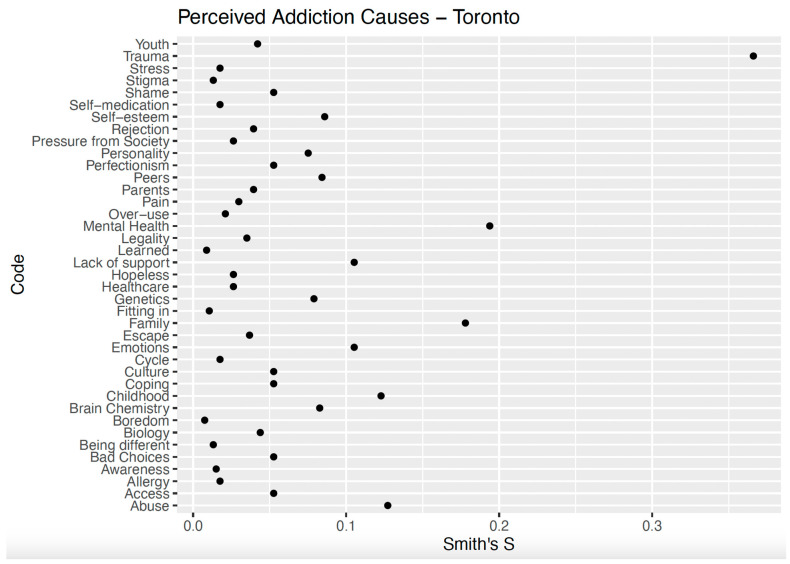
Smith’s S scores for perceived causes of addiction in Toronto.

In Toronto, the salient terms for the Canadian sample included *trauma* (Smith’s S = 0.37), *mental health* (Smith’s S = 0.19), *childhood* (Smith’s S = 0.12), *family* (Smith’s S = 0.18), *abuse* (Smith’s S = 0.13), and emotions (Smith’s S = 0.11; Figure 2). The Canadian responses describing addiction were a combination of open responses and lists, with many themes overlapping in the same response.

*Trauma* (Smith’s S = 0.37) was described as a key antecedent to addiction. To illustrate, Jessica explains that addiction is caused by “Depression, trauma. I feel like if it runs in your family bloodline, that helps. Your brain has a big part of it. Impulse. You have more sporadic impulses medically in your brain. I’ve learned that. Just different personalities. Like I said, lack of understanding and boredom.” Others felt that trauma was the sole cause. For example, Maria stated, “I think that addiction is a symptom of trauma.” She then said, “I mean, I guess it could also be like brain chemistry. But most of the time, I feel like it’s unresolved trauma or untreated mental health.” Here, we can see that participants perceive addiction as a consequence of the use, and eventual misuse, of substances to manage the psychological/emotional consequences of trauma history. This may be exacerbated by the presence of neurochemical or dispositional vulnerabilities, but the element of trauma is seen as essential.

*Mental health* (Smith’s S = 0.19) issues were often mentioned as a secondary cause of addiction. Anna is an example of this. She said,

“So, I think it’s just the unreal expectations that society has toward women, especially mothers, and like to be the perfect mother to look perfect to have a job, plus do all the things for your children, like extracurricular activities, to juggle that perfect life. And then, you know, when you have mental health issues like that, that are also, you know, it also, I just, I think mental health causes you to have, especially like postpartum depression or something like that.”

Similarly, Diana stated,

“Oh, that’s a real open-ended question. It could be anything from childhood trauma, abuse, mental illness. It could be physical pain from accidents. It could be homelessness or home insecurity. It could be just neglect in general. All of those. There may be even more. It could be systematic racial problems that they’ve faced or economic problems. A lot of it is all integrated. I don’t think there’s one or the—I don’t find one is without another. I think that they’re all somehow intertwined.”

Overall, addiction was perceived to be caused by various external stressors (e.g., social expectations, trauma, financial/housing insecurity, discrimination) when combined with individual vulnerabilities, specifically, mental illness.

*Childhood* (Smith’s S = 0.12) was often mentioned in the middle or toward the end of a list or excerpt but was mentioned frequently by participants. For example, Emily gave a long response regarding what she thinks causes addiction, and childhood appeared toward the end of her statement:

“Oh, what do we think causes it? You know, I’m not really of the opinion that you have to have some sort of like, trauma, to end up a drug user. You know, I think even just something so simple as bad choices can put you in a situation where you’re doing a drug that you have to do every day, even though that’s not something that you ever thought you would end up doing, you know, what I think causes it, you know, being a kid being dumb, you know, going out, being with different people, you know, everything could put you in a situation where you’re, you know, maybe going to do drugs where you normally wouldn’t? What makes you end up a habitual drug user? I think choosing the wrong drug too many times.”

Um, I think for some people, you know, maybe they have a really shitty upbringing, and, you know, or they’re living on the street. And these would be all good reasons to want to be high every day all day long. And I don’t think it’s like that for everybody. I think some people really are just functioning addicts. Yeah, sort of late getting high on their time off and they’ve picked something that you know, maybe it wasn’t a good pick.”

In this cultural context, childhood is perceived as contributing to addiction through both external (e.g., traumatic, unstable, and/or neglectful environments) and internal (e.g., being naive, lack of understanding, impulsivity) processes.

A specific feature of childhood and early life, one’s *family* (Smith’s S = 0.18), was described as a key contributor to addiction, given the control that caregivers have over one’s early developmental experiences and environment. Esther combined the themes of family and peers, but this excerpt showcases how family was more salient: “I think a lot of it has to do with your upbringing…Falling into the wrong crowd. If you say, for example, move to another country, city, or neighborhood, you fall into the wrong hands.” Annika also mentioned family as a salient cause of addiction,

“Well, yes, I mean, it starts as you know, like, you just don’t even think that because you grow up having it around, or people do or for me, personally, but it’s always around, and you don’t realize that you use it when you’re, you know, I’m also a smoker. So I was reading, you know, a cigarette, you can use when you’re happy when you’re sad when you’re mad like it. It’s the cure for everything.”

*Abuse* (Smith’s S = 0.13) was another salient cause of addiction. According to Leah, addiction is caused by “Grief, being abused, not feeling accepted, being different.” Similarly, Carla mentioned abuse as an important cause of addiction: “I think it comes from trauma and abusive relationships as well. Bad relationships with your parents feeling like you’re an outcast.” Within these two cultural domains (family and abuse), we can see that participants view familial and romantic relationships as playing a critical role in addictive processes, through the modeling of substance use and misuse as well as the application of trauma and stress (e.g., abuse, neglect).

*Emotions* (Smith’s S = 0.11) were mentioned as a cause of addiction by two participants. When asked what she thought caused addiction, Serena’s first response was, “Um, I think a lot of addicts the way it begins is typically some sort of emotional trigger that they don’t deal with. And then they kind of push aside and think that they’re okay.” Another participant, Leah, simply said “grief.”

#### 5.1.3. London

**Figure 3:**
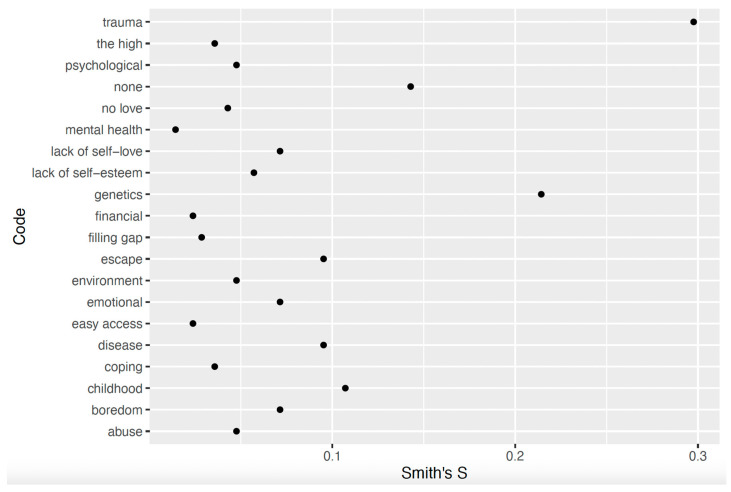
Smith’s S scores for perceived causes of addiction in London.

The salient terms for the London sample included *trauma* (Smith’s S = 0.30), *genetics* (Smith’s S = 0.21), *childhood* (Smith’s S = 0.11), and *none* (Smith’s S = 0.14). Figure 3 displays Smith’s S scores.

*Trauma* (Smith’s S = 0.30) was mentioned early in participants’ lists and often. For example, Elizabeth mentioned trauma first:

“In my opinion, trauma, behaviors copied, learned from what you witness as a child, especially around the age of seven to eight when you’re more likely to remember your life. I know for me it is the type of counseling I did and rehabilitation that I was in and what I’ve learned about myself, I was able to change some behaviors and recognize that and change those.”

For Annabelle, trauma was the sole cause of addiction: “What I noticed, the pattern was that there was always some sort of trauma. It always starts—A lot of people think—there’s this stereotype that people who take drugs, they’re homeless, they’ve caused this on themselves or whatever. A lot of the time, it actually started when they were younger and something traumatic happened to them. Even the idea, because I always thought there was this idea of like when you start using cannabis and then you want to try something new.”

Overall, it was clear that participants perceived trauma as a key precipitator of substance misuse and addiction, characterizing substance misuse as a strategy to manage the psychological/emotional consequences of trauma (especially trauma in childhood).

*Genetics* (Smith’s S = 0.21) was often mentioned first by participants but fewer times than trauma. From Charlotte’s perspective,

“That’s a really tricky question. For me personally, I think it was hereditary. My father and my brother are both alcoholics, so I think I have the addictive gene. Personally, I didn’t believe that I could be an alcoholic because I was female, and I didn’t think that women could be alcoholics. I think it can also come down to circumstance as well in terms of overuse can just cause you to become addicted to that.”

Diane also mentioned genetics first but was one of three participants who did so: “In my opinion, I think you are born with it to a certain degree, but I think life traumas contribute to it and a need to escape reality.” While the role that genetics and inheritance play in addiction was clearly salient for our participants (i.e., patterns of primacy for this factor across participants’ responses), it tended to be mentioned as precipitating addiction when in conjunction with other factors (e.g., traumas, childhood environment, frequent or habitual use).

*Childhood* (Smith’s S = 0.11) was mentioned by only two participants. To illustrate, Judy described childhood as a primary cause:

“I think past life, like childhood or really what’s happened to you in your life. You want to forget. Do you understand? Forget things or to cope with stuff that’s going to happen.”

For these two participants, childhood seemed to be perceived as a critical period wherein other risk factors (e.g., trauma, social influence via family or peers) were particularly likely to lead to addiction.

## 6. Discussion

This study aimed to assess cultural domains of addiction among women in recovery for substance misuse from three regions—Delhi, London, and Toronto—where rates of opioid misuse are currently on the rise. Specifically, we used free-listing and interview methods to elicit cultural models of the causes of addiction. The most notable differences emerged between participants in Delhi and those in Toronto and London. In Delhi, women emphasized pleasure and peer influence as primary causes of addiction, whereas participants in the Western sites more often cited trauma and childhood experiences. These differences may stem in part from age: women in Delhi were substantially younger and appeared to begin substance use recreationally with peers. Other research with adolescents and young adults similarly finds that peers are particularly influential, and that peer pressure and modeling of substance use are associated with an increased risk of escalating substance use [51] and addiction [52,53]. In contrast, the older participants in Toronto and London were more likely to frame addiction through the lens of early life experiences, which may have shaped their cultural models of addiction causation.

Additionally, trauma as a model of addiction is prominent in Western psychology, which may contribute to the differences observed between the two Western contexts and the Delhi site. Indeed, research conducted in Western contexts, including Canada [54] and the USA [55], finds that traumatic experiences during childhood are perceived to cause addiction to substances like alcohol and heroin. In Toronto and London, therapeutic approaches were largely trauma-informed; recovery coaches and therapists commonly framed substance misuse as a trauma response, reinforcing that model in both individual and group therapy. Research finds that trauma-informed approaches to addiction treatment are common and effective in UK [56] and Canadian [57] contexts. In Delhi, however, providers typically cited stress and biological factors as causes of addiction, making it unclear whether or how treatment practices there contributed to participants’ emphasis on pleasure and peer influence. One study conducted with adolescents (15–21 years) receiving treatment for addiction in Mumbai found that participants emphasized the hedonic (e.g., elatedness, relief from boredom, euphoria) and relational (e.g., self-confidence, feeling accepted by friends) outcomes they experience when under the influence of substances [58]. More research is needed to elucidate the mechanisms of transmission of addiction causes in these groups and to explore the extent to which specific cultural practices (e.g., therapeutic approaches, local norms and practices) may give rise to different conceptualizations of the causes of addiction.

Despite these differences across cultural groups, several shared characteristics were observed across all sites. Overall, participants’ narratives highlighted the presence of cultural models of addiction that attributed causes to psychological factors, specifically the use of substances to self-medicate and alleviate negative psychological states (e.g., “stress”, “mental health”, “loneliness”, “emotions”) and to promote positive psychological states (i.e., “pleasure”), relational factors (e.g., “peers”, “family”, “abuse”), developmental factors (e.g., “childhood”), and biological factors (i.e., “genetics”). This is consistent with other research into perceptions of addiction, specifically, the causes of addiction, in the USA (where psychological, relational, developmental, and biological factors are endorsed as causes) [15,59], Brazil (where psychological, developmental, and relational but not biological causes are endorsed) [60], and Iran (where environmental and relational factors are emphasized) [61].

The cultural models of addiction shared by our participants all included psychological causal attributions, reflecting the shared belief that escalating substance use and addiction were the result of using substances to manage negative or distressing mental states. In all three contexts, the mental distress precipitating addiction was primarily described as being caused by external factors. For example, women in Delhi used the word “tension” to describe the mental distress that causes addiction; while “tension” as a construct captures many experiences of psychological distress in South Asian samples, what these descriptions have in common is that they are seen as a psychological response to an external stressor (e.g., poverty, loss of a loved one, pressure to meet social expectations; [62,63]). Similarly, emphasizing the relationship between external stressors and internal, psychological states, the mental health issues perceived to make one vulnerable to developing an addiction among participants in Toronto and London were described as outcomes of conditions like financial or housing insecurity, the social expectations placed on women and mothers, and traumatic experiences. The links between external stressors, especially deprivation, poor mental health, and substance use, are well established in the literature [64,65]. Furthermore, research supports the role that trauma, both direct (experiencing or witnessing trauma) and indirect (learning about trauma secondhand), plays in vulnerability to substance and behavioral addictions [66].

Cultural models of addiction shared by women in Toronto and London position this health condition as developmentally sensitive, with early development and childhood playing critical roles in the establishment of patterns of substance misuse. In both contexts, participants emphasized the role of negative experiences during childhood, neglect, abuse, exposure to drug use, or violence in one’s early environment in increasing the risk of developing addiction. Participants in Toronto highlighted the naivety and impulsivity of childhood as key contributors to substance use and addiction. The role of adverse childhood experiences (ACEs) on the development of substance use disorders later in life is supported by a large body of empirical work [67,68]; to illustrate, in a sample of adults in the USA a study found that, compared to participants with zero reported ACEs, those who reported experiencing five or more categories of ACEs (e.g., emotional abuse, physical abuse, emotional neglect, mental illness in household, incarcerated household member) were 7 to 10 times more likely to report substance use problems, including addiction [69].

In addition, both women in Toronto and Delhi emphasized the relational causes of addiction—family and abuse in Toronto, and peers and loneliness in Delhi—highlighting the critical role of social relationships in shaping both addiction pathways and recovery trajectories [38,70,71]. Cross-culturally, research indicates that social support is a protective factor for recovery, with differential effects on men and women [70,71]. In a Canadian study conducted with people who injected drugs, findings revealed that social support decreased the risk of non-fatal drug overdoses in women but not men [71]. Other research conducted among women shows similar trends: access to social networks and perceived social support bolsters mental health and lowers drug use in recovery [72,73]. Taken together, these findings underscore the importance of addressing mental health alongside substance use disorders while also addressing local, sociocultural factors that give rise to addiction.

Finally, unique features of cultural models shared by our participants emerged, such that hedonistic causes of addiction (e.g., pleasure, enjoyment, good sensations; [60]) were only salient for participants in Delhi. The role of positive psychological or emotional states in shaping substance use, specifically, curiosity, enjoyment, and relaxation, has been identified in other research with participants living in India, i.e., Delhi, Goa, and Kerala [74,75]. Another unique feature was the mention of biological causes of addiction, specifically, a genetic predisposition or sensitivity; this was only salient for participants in London.

This study was limited in that it did not compare women’s perceptions of addiction to those of men, which could shed light on gender differences in cultural models. Furthermore, while this study focused primarily on addiction, terms like “drug” and “substance” are also culturally diverse. When asked about commonly used drugs in their communities, some participants quickly cited “alcohol,” while others asked if alcohol counted as a drug. In retrospect, we could have first invited participants to define “drug” and list examples from their communities. Using this inductive approach for key terms in substance use research fosters more culturally competent data and shared understanding between participants and investigators.

Despite potential limitations, the findings presented here underscore the social processes that participants in the three cultural contexts view as key in shaping addictive processes, such as the role of peers and family members who model and normalize substance (mis)use or the role of harmful or traumatic interpersonal stressors (particularly in childhood). These findings contradict mainstream views of addiction and recovery, which are often conceived as individual conditions or imply an internal locus of control. For instance, “recovery” is inconsistently defined across clinical research, and instruments like the Recovery Capital Scale [76] reflect Western social and biomedical values that focus on the individual [77]. Statements such as “I have an intimate partner supportive of my recovery process” may imply a degree of control over personal relationships that is not universally applicable, potentially leading to culturally biased assessments. This contextualization of recovery as a primarily individual process also fails to capture the role that relationships (peers, families, etc.) play in both addictive and recovery processes. A culturally competent understanding of addiction and recovery thus requires that participants help define these terms themselves, especially in diverse cultural contexts. 

In conclusion, this study underscores the importance of incorporating cultural models into both the treatment and research of drug misuse. Although the focus here was primarily on perceptions of addiction, it is crucial to recognize that other terms and symbols associated with substance use also carry meaning and can shape individuals’ experiences as they navigate recovery. By employing an inductive approach to explore the constructs surrounding drug use among people in recovery, researchers can gain insight into how interventions are interpreted and integrated into individuals’ existing internal frameworks. This study also offers a more nuanced approach to conceptualizing and measuring “culture” in addiction research, a term that is often used interchangeably with race and ethnicity or left insufficiently defined [77,78,79]. With these contributions in mind, further global research is essential to better understand patterns of drug use and the sociocultural factors influencing continued use and recovery, particularly as the gender gap in drug use narrows in regions where access to criminalized substances is increasing [1].

## Data Availability

Data is unavailable due to privacy restrictions.
